# Drosophila eiger Mutants Are Sensitive to Extracellular Pathogens

**DOI:** 10.1371/journal.ppat.0030041

**Published:** 2007-03-23

**Authors:** David S Schneider, Janelle S Ayres, Stephanie M Brandt, Alexandre Costa, Marc S Dionne, Michael D Gordon, Eric M Mabery, Madeleine G Moule, Linh N Pham, Mimi M Shirasu-Hiza

**Affiliations:** Department of Microbiology and Immunology, Stanford University, Stanford, California, United States of America; Massachusetts General Hospital and Harvard Medical School, United States of America

## Abstract

We showed previously that *eiger,* the *Drosophila* tumor necrosis factor homolog, contributes to the pathology induced by infection with Salmonella typhimurium. We were curious whether *eiger* is always detrimental in the context of infection or if it plays a role in fighting some types of microbes. We challenged wild-type and *eiger* mutant flies with a collection of facultative intracellular and extracellular pathogens, including a fungus and Gram-positive and Gram-negative bacteria. The response of *eiger* mutants divided these microbes into two groups: *eiger* mutants are immunocompromised with respect to extracellular pathogens but show no change or reduced sensitivity to facultative intracellular pathogens. Hence, *eiger* helps fight infections but also can cause pathology. We propose that *eiger* activates the cellular immune response of the fly to aid clearance of extracellular pathogens. Intracellular pathogens, which can already defeat professional phagocytes, are unaffected by *eiger*.

## Author Summary

We show that the gene *eiger,* which is the sole tumor necrosis factor homolog in the fruit fly, can play opposing roles in the fly's response to infections. Sometimes *eiger* contributes to the disease induced by an infection, while at other times it is required to fight an infection. Commonly, the fly's immune response is described as dividing microbes into two groups with Gram-positive bacteria and fungi lying in one group and Gram-negative bacteria lying in the other. Pathogenic bacteria can also be divided into two groups based on their behavior in *eiger* mutant flies, but these two groups differ from past descriptions. *eiger* tends to be required for the innate immune response against extracellular pathogens but tends to cause pathology during an infection with an intracellular pathogen. We suggest that *eiger* is required for innate immune responses that are effective at fighting extracellular pathogens but are wasteful or simply ineffective when fighting intracellular pathogens. We show here that the fly immune response is more complex than previously recognized and suggest new directions for studying pathogenesis in addition to innate immunity in the fly.

## Introduction

The fruit fly has four main immune mechanisms to fight circulating microbes. These mechanisms include secreted antimicrobial peptides (AMPs), melanization, clotting, and phagocytic hemocytes [1−4]. The most deeply studied of these mechanisms, AMP secretion, is controlled by the *Toll* and *imd* pathways, which regulate the transcription of AMP genes in the fat body. Signaling through the *Toll* and *imd* pathways is activated by microbial elicitors; for example, the receptor peptidoglycan receptor protein LC, one receptor that triggers *imd* signaling, is activated by diaminopimelic acid–containing peptidoglycan [5,6]. This material is found on most Gram-negative bacteria as well as some Gram-positive bacteria.

Mutations affecting the *Toll* and *imd* pathways severely immunocompromise flies. Although *Toll* and *imd* mutant flies get sick and die from infections, this does not resemble many infectious processes in healthy humans. For example, an *imd* mutation essentially turns flies into a passive culture medium for *Escherichia coli;* the bacteria grow 1,000-fold in 24 h [7,8]. Immunocompromised infected flies likely die from the enormous load of bacteria that can reach levels of more than 1% of the mass of the fly. Death from massive numbers of microorganisms can happen in humans, particularly in immunocompromised patients; however, the infectious agents that are responsible for the greatest mortality in humans—*Mycobacterium tuberculosis,* HIV*, Plasmodium falciparum,* and diarrhea-inducing microbes—do not work in this manner. Instead, relatively small numbers of these infectious agents cause various pathologies that lead to death. To study microbial pathogenesis in the fly, it is necessary to follow microbes that cause disease in wild-type flies.

Pathogens are different from nonpathogenic bacteria and simple molecular elicitors of innate immunity, and thus the results we see from experiments with pathogens will be different than those observed for simple elicitors like E. coli and Micrococcus luteus. For one thing, pathogens can override the strong immune defenses of the fly; this causes disease. In flies, as in humans, there is more than one type of disease that results from infection. Pathogens have been observed to kill the fly in at least four different ways: first, overactivation of the *Toll* or *imd* pathways can be pathogenic [9,10]; second, Vibrio cholera fed to flies kills the fly through the secretion of toxins that presumably cause physiological changes to the gut [11]; third, M. marinum causes a wasting disease in flies [12]; and fourth, Salmonella typhimurium secretes effectors through its type III secretory system that increase the pathogenicity of the microbe. The fly gene *eiger,* the fly's sole tumor necrosis factor homolog, is implicated in causing pathology during this infection because S. typhimurium–infected *eiger* mutant flies live longer than infected wild-type flies [13]. It appears that there are many physiological routes that can lead to death following infection.

We have begun to try to understand these physiological routes to death by analyzing the role played by *eiger* in a variety of infections. The *eiger* mutation divided our group of pathogens into two unanticipated groups. The first group of microbes kills *eiger* mutants more rapidly than wild-type flies; this group includes a fungus as well as both Gram-positive and Gram-negative bacterial species. The second group of microbes kills *eiger* mutants at the same rate or more slowly than wild-type flies; this group includes a Gram-positive and a Gram-negative bacterial species and a mycobacterium.

These results cut across the previous descriptions of *Drosophila* immunity, which groups microbes according to their gross physical characteristics [2,3]. The most obvious way to explain the grouping of these pathogens in *eiger* mutants is not by grouping microbes according to their Gram-staining properties or kingdom, but rather by their pathogenesis mechanisms; the first group of microbes consists of extracellular pathogens while the second group contains microbes that can grow within professional phagocytes. *eiger* appears to play a role in innate immunity in fighting extracellular pathogens but plays a role in driving pathogenesis when fighting some intracellular pathogens. This study demonstrates that the examination of real pathogens and the use of outputs other than AMP transcription leads to the discovery of unanticipated immune pathways and reveals new complexities in the *Drosophila* immune system.

## Results/Discussion

To determine the role *eiger* plays in fighting microbial pathogens, we tested a collection of Gram-positive, Gram-negative, and fungal pathogens that could cause either intracellular or extracellular infections (Table 1). Our goal was to test a broad group of microbes that used different virulence mechanisms and were recognized by different innate immunity pathways.

**Table 1:**
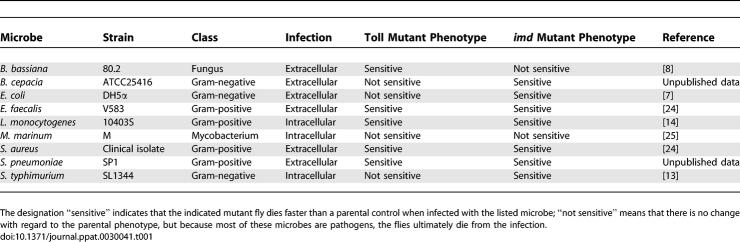
Microbial Strain List

**Figure 1:**
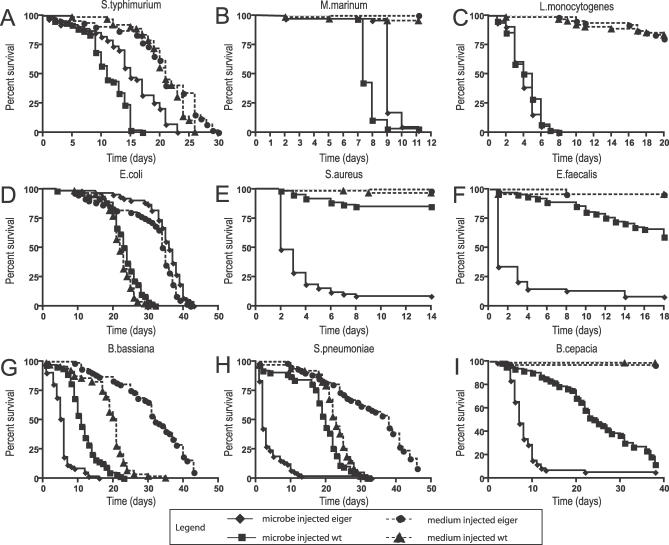
Survival of *eiger* Infected Flies Week-old male flies were infected with pathogens and survival was monitored daily. (A) *S. typhimurium; (*B) *M. marinum,* (C) *L. monocytogenes,* (D) *E. coli,* (E) *S. aureus,* (F) *E. faecalis,* (G) *B. bassiana,* (H) *S. pneumoniae,* (I) B. cepacia. Circles indicate medium-injected *eiger;* diamonds, microbe-injected *eiger;* squares, microbe-injected parental; triangles, medium-injected parental. Medium injection is indicated by a dotted line, while microbe injection is indicated by a solid line. Statistical significance was determined using log-rank analysis. The infected *eiger* and wild-type curves in (A), (B), and (E–I) are significantly different with *p* < 0.0001 as determined by log-rank analysis.

We tested a heterozygous combination of two *eiger* null alleles *(w^1118^; egr^1^/egr^3^)* and compared this to an isogenic *w^1118^* parental strain (Figure 1). *eiger* mutants died much faster than wild-type flies when challenged with *Beauveria bassiana, Burkholderia cepacia, Enterococcus faecalis, Staphylococcus aureus,* and Streptococcus pneumoniae (Figure 1). The members of this group of microorganisms are very different from each other and include Gram-positive and Gram-negative bacteria and a fungus. The smallest effect on survival was a 50% reduction in the mean time of death for B. bassiana infections. The effect was strongest with *eiger* mutants infected with S. aureus and *E. faecalis;* whereas the chosen doses were nonpathogenic to wild-type flies (i.e., they died at the same rate as media-injected controls), *eiger* mutants died within 2 d. The one common characteristic of this group is that these microbes are all expected to produce extracellular infections.

*eiger* mutant flies did not die faster when infected with the three facultative intracellular pathogens we tested (Figure 1). We reported previously that *eiger* mutants live longer than wild-type flies when infected with S. typhimurium. This result led us to suggest that *eiger* activity could be deleterious for the fly and was a cause of pathology. We found that M. marinum–infected *eiger* mutants also live longer than infected wild-type flies. Finally, *eiger* mutants and wild-type flies survive exactly the same amount of time when infected with Listeria monocytogenes.

We tested E. coli as a nonpathogenic control (Figures 1 and S1). We define a pathogen as a microbe that increases the death rate of infected flies as compared to a control fly injected with medium. This microbe was chosen because it played a major role in characterizing the immune response of the fly. E. coli is normally a good inducer of innate immune responses when injected into the fly but will only cause disease in flies missing the *imd* pathway. E. coli injection does not kill *eiger* mutants or wild-type flies. This suggests either that *eiger* does not play a role in fighting a nonpathogenic infection or that *eiger*'s role is redundant and therefore undetectable by this assay.

**Figure 2:**
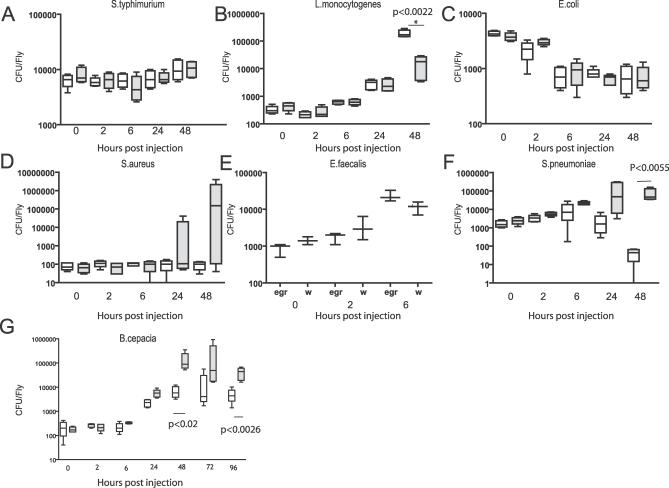
Bacterial Growth in *eiger* Mutants Week-old male flies were infected with pathogens, and flies were collected at 0, 2, 6, 24, and 48 h, if there were survivors. Live flies were homogenized and plated: (A) *S. typhimurium;* (B) *L. monocytogenes;* (C) *E. coli;* (D) *S. aureus;* (E) *E. faecalis;* (F) *S. pneumoniae;* (G) B. cepacia. Data are plotted as box plots with whiskers. White bars indicate the parental *w^1118^* line; gray bars, *w^1118^; eiger^1^/eiger^3^* mutants. Statistical significance was calculated using two-tailed nonparametric *t*-tests.

We next decided to determine whether *eiger* mutant flies were killed faster by extracellular pathogens because of increased bacterial growth or increased pathogenesis by monitoring bacterial proliferation in infected flies (Figure 2). As before, bacteria were injected into age-matched male wild-type parental or isogenic transheterozygous *eiger* mutant flies. Flies were collected and homogenized following bacterial challenge to measure bacterial loads.

Of the four bacterial species that showed increased virulence in *eiger* mutants, two—B. cepacia and S. pneumoniae—had statistically significant increased growth rates in *eiger* mutants. There was no clear effect on E. faecalis growth rates in *eiger* versus wild-type flies. S. aureus showed a large variation in bacterial numbers toward the end of the infection that suggested a trend toward increased growth; for example, the highest levels of bacteria found in *eiger* mutants were 10,000 higher than those seen in wild-type flies. However, because the variation was so great in *eiger* mutants, the difference between wild-type and *eiger* mutants was not statistically significant. We suggest that S. aureus might not cause synchronous infections and thus that some flies succumb to infection rapidly and have high numbers of bacteria while others take longer to die and maintain lower levels of bacteria. This would result in a huge range of bacterial titers in a group of injected flies. E. coli were cleared at comparable rates in *eiger* and wild-type flies. B. bassiana was not tested because we do not have a good quantitative method of measuring fungal growth. In summary, these bacterial growth measurements show that *eiger* mutants are unable to limit the growth of two and possibly three of the four extracellular pathogens tested. This experiment does not rule out the possibility that *eiger* mutants suffer greater pathological effects of infection but does suggest that *eiger* in a wild-type fly plays a role in reducing the numbers of extracellular microbes.

The proliferation of facultative intracellular pathogens did not increase in *eiger* mutants. As we published previously, S. typhimurium levels remained constant in an *eiger* homozygote as compared to an isogenic wild-type fly [13]. L. monocytogenes numbers decreased significantly in *eiger* mutants with respect to wild-type flies even though both wild-type and mutant flies died at the same rate. Our interpretation is that *eiger* is somehow helpful for the growth of *Listeria*. We did not measure the growth rate of M. marinum.

There is no simple interpretation, using *Toll* or *imd* signaling, that can explain the difference in sensitivity of *eiger* mutants to the tested pathogens. If the *eiger* mutation resulted in decreased *Toll* signaling, then we would expect *Listeria* to kill the flies rapidly as has been reported [14]. Likewise, a reduction of *imd* activity would immunocompromise the flies to *S. typhimurium. imd* mutants are very sensitive to S. typhimurium and will die within 24 h when infected with as little as a single colony-forming unit [13]. Furthermore, if *imd* signaling was affected, we would expect flies to become sensitive to *E. coli,* which they do not [7]. We are forced to conclude that the strong effects of *eiger* on the pathogenesis caused by extracellular microbes are not caused by a reduction in signaling through the *Toll* and *imd* pathways.

**Figure 3:**
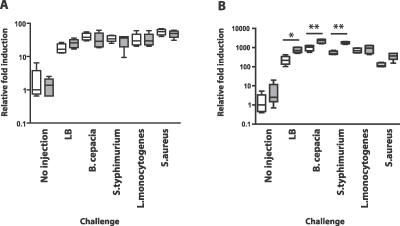
Antimicrobial Gene Expression Differences between *eiger* Mutants and Wild-Type Controls Flies were injected with an LB control, *B. cepacia, S. typhimurium, L. monocytogenes,* or *S. aureus* or left uninjected. RNA was harvested after a 6-h incubation at 29 °C, and quantitative RT-PCR was used to assay drosomycin (A) and diptericin (B) gene expression levels relative to a ribosomal protein 15a control. White bars indicate the *w^1118^* parental control; grey bars, *w^1118^; egr^1^/egr^3^* One asterisk indicates *p* < 0.01; two asterisks, *p* < 0.001 using Tukey's multiple-comparison test following one-way ANOVA. Unmarked parental/mutant pairs do not differ in a statistically significant manner. No differences were seen with drosmycin expression between mutant and parental lines, whereas *eiger* mutant flies transcribed 2- to 3-fold more diptericin (during a 150- to 1,100-fold induction compared with uninjected flies) than did their parents, when challenged with LB, *B. cepacia,* or *S. typhimurium.*

To test the hypothesis that *Toll* and *imd* signaling are not grossly reduced by a mutation in *eiger,* we measured the induction of AMP transcription in *eiger* and parent strains (Figure 3). Flies were challenged with *S. typhimurium, B. cepacia, L. monocytogenes,* and *S. aureus* as well as an LB control, and these conditions were compared to expression levels seen in uninfected flies. We chose this subset of bacteria because it included both Gram-positive and Gram-negative examples of intracellular and extracellular pathogens. *Toll* signaling was monitored by following *drosomycin* gene induction using quantitative reverse transcription (RT)-PCR, although we note that there are no perfect *Toll* responsive genes and *drosomycin* is responsive to both *Toll* and *imd* signaling [15]. To measure the output of the *imd* pathway, we monitored the expression of the *diptericin* gene. No statistically significant changes in *drosomycin* transcription were seen between parental and eiger mutant flies. Some conditions caused a relatively small, but statistically significant increase in *diptericin* transcription in *eiger* mutants. We do not understand how the slightly higher levels of AMP transcription found in *eiger* mutants might affect the *eiger* phenotype. The important point is that *eiger* mutants did not have lower levels of AMP gene expression than did wild-type flies, demonstrating that the *eiger* phenotype is not caused by reduction in *Toll* or *imd* signaling.

**Figure 4:**
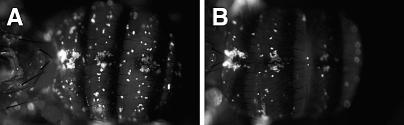
Phagocytosis in *eiger* Mutants Week-old male flies were injected with FITC-labeled dead S. aureus and allowed to phagocytose the particles and then were injected with Trypan blue to quench extracellular fluorescence. The dorsal abdomen of the fly was photographed under epifluoresence optics. Anterior is on the left. (A) Wild-type fly *(w^1118^);* (B) *eiger* mutant fly *(w^1118^; egr^1^/egr^3^).* Bright spots indicate hemocytes that have phagocytosed the *S. aureus.* The pictures are representative of the 15 flies examined for the experiment.

As *Toll* and *imd* signaling, and thus the majority of the antimicrobial peptide response in the fly [15], does not seem to be responsible for changes in *eiger* mutants, we next probed the cellular immune response (Figure 4). To determine whether *eiger* mutations might affect hemocyte function, we monitored phagocytosis in *eiger* mutants. Fluorescein isothiocyanate (FITC)-labeled S. aureus were injected into the hemocoel of wild-type or *eiger* mutant flies and the injected flies were given 1 h to phagocytose the particles. S. aureus was chosen because FITC-labeled dead S. aureus are commercially available and we predicted that *eiger* alters the ability of hemocytes to fight this pathogen. Trypan blue was injected into the hemocoel of the flies following the 1-h incubation period. This dye quenches the fluorescence of extracellular FITC but allows phagocytosed particles to fluoresce brightly [16]. The flies were examined around the dorsal anterior abdomen because hemocytes tend to gather in this area. Changes in phagocytosis can be seen by changes in the numbers of phagocytosing cells or by the fluorescence intensity. Both of these characteristics appear altered in *eiger* mutants compared to parental flies, suggesting that *eiger* mutant hemocytes were either reduced in number or had reduced phagocytic activity, or both.

We note that uninfected *eiger* mutants live longer than wild-type flies. This raises the possibility that the increased survival of *eiger* mutant flies after infection with M. marinum or S. typhimurium is simply a consequence of general increased longevity. We argue that this possibility is unlikely, because it assumes that the cause of death due to old age is the same as the cause of death by infection; this is not observed in other animals and it is not supported by examination of published microarray studies of flies dying from infection or old age [12,17,18]. Instead, we argue that *eiger* is a driver of pathogenesis. If *eiger* is indeed required to fight infections but can also cause pathology, then the phenotype we observed for a given infection is likely the sum of the positive and negative effects of *eiger*. Regardless of the interpretation of the effects of *eiger* on S. typhimurium and M. marinum, the important result remains that *eiger* mutations divide the fly's response to pathogens into two groups.

Why does *eiger* affect different microbes in different ways? We argue that E. coli is removed from flies so rapidly and via so many mechanisms that the effects of *eiger* cannot be measured easily because of redundancy. In contrast, during an infection with a real pathogen, it may be easier to measure changes in immunity because the fly is fighting hard for survival and its immune mechanisms are not acting in a redundant fashion. Extracellular pathogens are clearly fought by the fly using *eiger*-dependent mechanisms because the loss of *eiger* results in a deeply sensitive phenotype. It is intriguing that flies lacking *eiger* are no worse at fighting intracellular pathogens. This suggests that these intracellular pathogens are normally immune to the effects of wild-type *eiger*. Intracellular pathogens like *M. marinum, L. monocytogenes,* and S. typhimurium use different virulence mechanisms for growing inside cells, but the common thread is that they can survive in professional phagocytes. We suggest that *eiger* function somehow alters hemocytes to increase their potency against microbes. The reduced ability of *eiger* mutant hemocytes to phagocytose S. aureus compared to wild-type hemocytes supports this hypothesis. We predict that this change in potency is effective against pathogens that grow extracellularly but not against pathogens that have already developed methods of defeating phagocytes.

We propose that where *eiger* signaling does not help fight infection, *eiger* can cause pathology. This *eiger*-induced pathology may be linked to the immune function of *eiger*—for example, the induced immune response may be energetically wasteful or directly toxic. Alternatively, *eiger*-induced pathology may be separable from the immune function—for example, *eiger* could cause something like muscle wasting in the fly, as has been suggested for tumor necrosis factor in vertebrates [19].

Genetic screens that monitored AMP synthesis have been productive and filled in the *Toll* and *imd* pathways but did not reveal *eiger* signaling [20,21]. As we have shown, the role *eiger* plays in innate immunity cannot be measured using a nonpathogenic microbe like E. coli. This study demonstrates that there are important immune mechanisms at work in the fly that are difficult to see using simple endpoints like antimicrobial gene expression; however, studies with microbes that can cause disease in wild-type flies—real pathogens—can reveal these physiologies.

## Materials and Methods

### Fly strains.

The wild-type parental strain used in all experiments is *white^1118^.* The *eiger* alleles *white^1118^;egr^1^* and *white^1118^;egr^3^* were kept as homozygous stocks and crossed to make heterozygous flies as required [22]. We used this heterozygous combination to reduce the probability that there were other mutations on the chromosome that affected pathogenesis. To control for variation in the flies, we infected only 5- to 7-d-old male flies.

### Bacterial strains.

Strains used are listed in Table 1.

### Pathogen culture conditions.

B. bassiana cultures were grown on malt agar at 29 °C for 2 wk or until a sufficient density was reached, and the cultures were allowed to sporulate. Anesthetized flies were shaken on the plates for 30 s to coat flies with spores. Flies were transferred to fresh vials and incubated at 29 °C for the duration of the survival experiment.

S. pneumoniae cultures were grown standing at 37 °C 5% CO_2_ in brain heart infusion medium (BHI) to an OD_600_ of 0.15, and aliquots were frozen at −80 °C in 10% glycerol. For infection, an aliquot of S. pneumoniae was thawed, diluted 1:3 in fresh BHI, and allowed to adjust at 37 °C 5% CO_2_ for 1.5 h. *E. coli, S. typhimurium, E. faecalis,* and S. aureus cultures were grown overnight in Luria Bertani (LB) medium at 37 °C. L. monocytogenes was grown standing overnight in BHI medium.*B. cepacia, L. monocytogenes*, *S. pneumoniae,* and S. typhimurium were grown standing, while *E. coli, E. faecalis,* and S. aureus were shaken. M. marinum was cultured standing at 29 °C in Middlebrook 7H9 broth supplemented with Middlebrook oleic acid–albumin–dextrose–catalase enrichment and 0.2% Tween. Then 50 nl of bacteria was injected at the following optical densities (OD_600_): *B. cepacia,* 0.0001–0.001;*E. coli,* 0.1*; E. faecalis,* 0.5*; L. monocytogenes,* 0.01*; M. marinum,* 0.05*; S. aureus,* 0.001*; S. pneumoniae,* 0.05*;* and *S. typhimurium,* 0.1*.*

### Injection.

Five- to 7-d posteclosion male flies were used for injection. The flies were raised at 25 °C, 65% humidity, on yeasted dextrose food. Flies were anesthetized with CO_2_. Injections were carried out with a pulled glass capillary needle. A picospritzer (Parker Hannifin, http://www.parker.com) was used to inject 50 nl of liquid into each fly with needles that were individually calibrated by measuring the size of the expelled drop under oil. Reproducibility was measured by determining the number of bacteria injected at time zero and can be seen in Figure 2. Injected flies were incubated 20 flies to a vial and placed at 29 °C, 65% humidity with the exception of B. cepacia and M. marinum. B. cepacia infections were performed at 18 °C in the dark, and humidity was not controlled in this experiment. This temperature was used because B. cepacia is so pathogenic that it is difficult to obtain sufficient survival at higher temperatures to observe changes in death rates. M. marinum infections were carried out at 25 °C, 65% humidity, in the dark.

### Survival curves.

Parental flies *(w^1118^)* and *w^1118^; eiger^1^/eiger^3^* mutants were injected with the microbe of choice or medium as a control. Sixty flies were assayed for each survival curve, and they were placed in three vials of 20 flies each. Death was recorded daily. Data were not censored. Survival curves are plotted as Kaplan-Meier plots, and statistical significance is tested using log-rank analysis using Prism software (http://www.prism-software.com). Kaplan-Meier plots are shown in Figure 1, and survival curves showing the variance in the data are included in Figure S1. All experiments were performed at least three times and yielded similar results.

### CFU determination.

Following challenge with microbes, six individual flies were collected at each time point. These flies were homogenized, diluted serially, and plated onto appropriate media (blood agar for S. pneumoniae, LB for all others). E. faecalis CFUs were determined by testing three groups of six flies for each time point. The data are plotted as boxes with whiskers. The median is indicated with a bold line. The boxes indicate the extent of the third and first quartiles, while the whiskers show the complete range of the data. Statistical significance was determined using nonparametric two-tailed *t-*tests. All experiments were performed at least three times and yielded similar results.

### Antimicrobial peptide gene expression.

Flies were injected with 50 nl of the indicated microbes or controls. Following injection, the flies were placed in vials containing yeasted dextrose food and incubated at 29 °C for 6 h. Groups of five flies were homogenized in TriZOL and stored at −70 °C until processed. RNA was isolated using a standard TriZOL preparation, and the samples were treated with DNase (Promega, http://www.promega.com). Quantitative RT-PCR was performed as described [23] previously using a Bio-Rad icycler (http://www.bio-rad.com) and the following primer sets: drosomycin 5′-gracttgttcgccctcttcg, drosomycin 3′-cttgcacacacgacgacag, drosomycin Taqman probe tccggaagatacaagggtccctgtg, diptericin 5′-accgcagtacccactcaatc, diptericin 3′-cccaagtgctgtccatatcc, and diptericin Taqman probe cagtccagggtcaccagaaggtgtg. The data shown in Figure 4 include six biological replicates of each treatment condition, and each data point was calculated as the mean of two technical replicates. This experiment was repeated once using a set of three biological replicates with similar results. The data are plotted as boxes with whiskers. The median is indicated with a bold line. The boxes indicate the extent of the third and first quartiles, while the whiskers show the complete range of the data.

### Phagocytosis assays.

Flies were injected with 50 nl of 1 mg/ml FITC-labeled S. aureus (Molecular Probes, http://probes.invitrogen.com) in water. The flies were allowed to phagocytose the particles for 1 h and then were injected in the thorax with approximately 250 μl of 4% Trypan blue: this quenches the fluorescence of extracellular bacteria but permits the phagocytosed particles to fluoresce. The wings of the flies were removed with iris scissors, and the flies were pinned with a minuten pin and photographed using epifluorescent illumination with a Leica MZ3 microscope fitted with an ORCA camera. Images were captured with Openlab (Improvision) software (http://www.improvision.com). The exposure was set such that the brightest images had a small number of saturating pixels. The experiment was repeated three times with at least five flies for each treatment.

## Supporting Information

Figure S1 Variance in Survival of *eiger*-Infected Flies Week-old male flies were infected with pathogens and survival was monitored daily. (A) *S. typhimurium;* (B) *M. marinum,* (C) *L. monocytogenes,* (D) *E. coli,* (E) *S. aureus,* (F) *E. faecalis,* (G) *B. bassiana,* (H) *S. pneumoniae,* and (I) B. cepacia. Circles indicate medium-injected *eiger;* diamonds, microbe-injected *eiger;* squares, microbe-injected parental; triangles, medium-injected parental. The mean is plotted, and error bars show the standard deviation from three groups of 20 injected flies.
(1.4 MB AI).

## Abbreviations

AMP: antimicrobial peptide
FITC: fluorescein isothiocyanate
RT: reverse transcription
