# Supplementary material for: Drosophila eiger Mutants Are Sensitive to Extracellular Pathogens
Source: PLoS Pathog. 2007 Mar 23;3(3):e41. doi: 10.1371/journal.ppat.0030041 (PMC1829408; doi:10.1371/journal.ppat.0030041)
Supplement: Supplementary file 1 [file ppat.0030041.sg001.pdf]

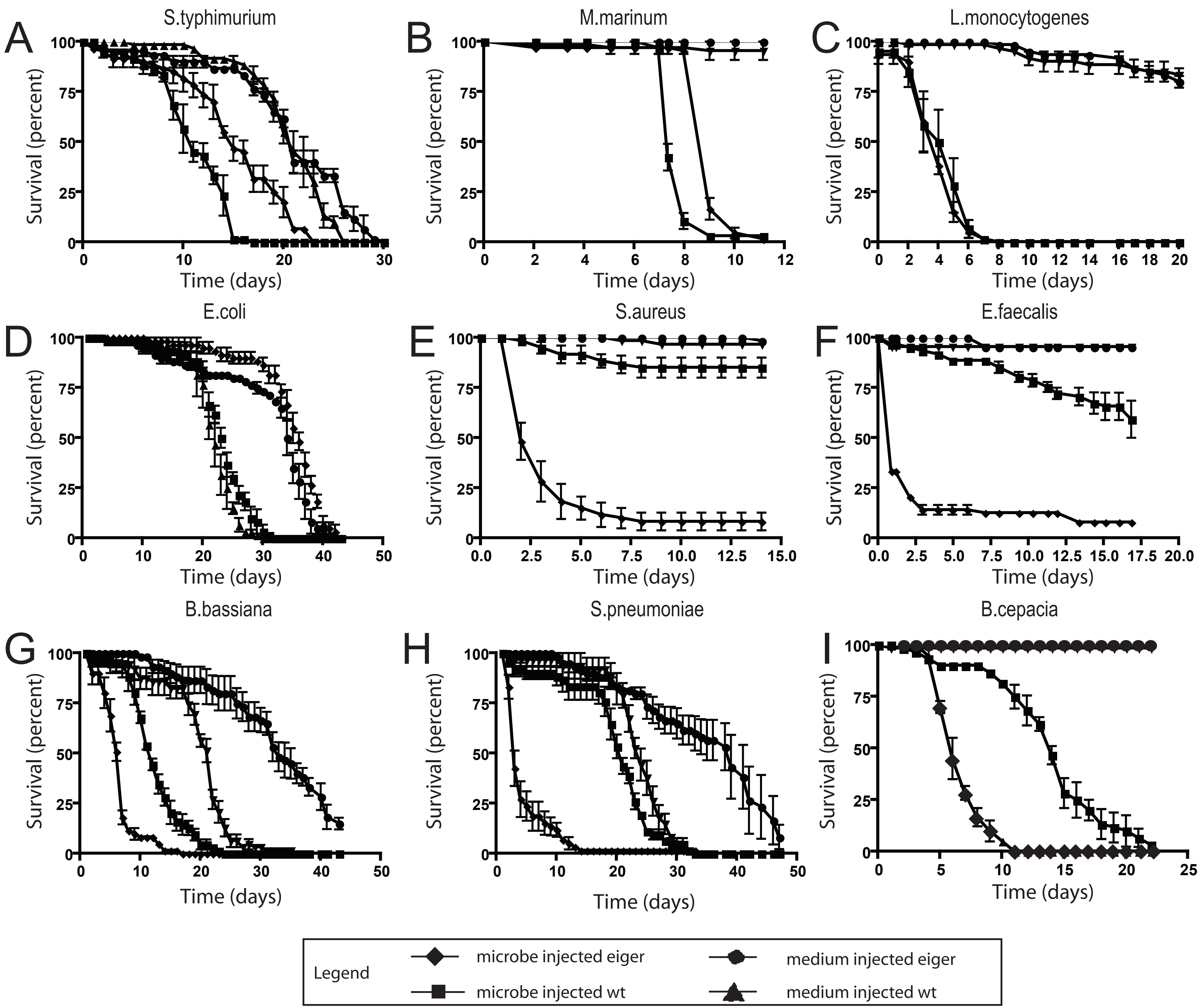
S.typhimurium
M.marinum
L.monocytogenes
E.coli
S.aureus
E.faecalis
B.bassiana
S.pneumoniae
B.cepacia
A
B
C
D
E
F
G
H
I
Survival (percent)
Time (days)
Legend
microbe injected eiger
microbe injected wt
medium injected eiger
medium injected wt
